# Can we enhance working memory? Bias and effectiveness in cognitive training studies

**DOI:** 10.3758/s13423-024-02466-8

**Published:** 2024-02-16

**Authors:** Jose A. Rodas, Afroditi A. Asimakopoulou, Ciara M. Greene

**Affiliations:** 1 Escuela de Psicología, Universidad Espíritu Santo, Samborondón, Ecuador; 2 School of Psychology, University College Dublin, Dublin, Ireland

**Keywords:** Working memory, Cognitive training, Executive functions, Fluid intelligence, Meta-analysis, Risk of bias

## Abstract

Meta-analyses have found that working memory (WM) can be improved with cognitive training; however, some authors have suggested that these improvements are mostly driven by biases in the measurement of WM, especially the use of similar tasks for assessment and training. In the present meta-analysis, we investigated whether WM, fluid intelligence, executive functions, and short-term memory can be improved by cognitive training and evaluated the impact of possible sources of bias. We performed a risk of bias assessment of the included studies and took special care in controlling for practice effects. Data from 52 independent comparisons were analyzed, including cognitive training aimed at different cognitive functions. Our results show small improvements in WM after training (SMD = 0.18). Much larger effects were observed when the analysis was restricted to assessment tasks similar to those used for training (SMD = 1.15). Fluid intelligence was not found to improve as a result of training, and improvements in WM were not related to changes in fluid intelligence. Our analyses did however indicate that cognitive training can improve specific executive functions. Contrary to expectations, a set of meta-regressions indicated that characteristics of the training programme, such as dosage and type of training, do not have an impact on the effectiveness of training. The risk of bias assessment revealed some concerns in the randomization process and possible selective reporting among studies. Overall, our results identified various potential sources of bias, with the most significant being the choice of assessment tasks.

**Supplementary Information:**

The online version contains supplementary material available at 10.3758/s13423-024-02466-8.

An important component of health is adequate cognitive functioning, which means that the mind is capable of understanding, interacting with, and adapting to the environment. Some mental abilities, such as intelligence, have also been found to be highly related to academic and professional success (Schmidt & Hunter, 2004; Strenze, 2007), an association which further accentuates the interest in restoring or even enhancing them. It is often argued that these functions are very difficult to modify, requiring cognitive rehabilitation techniques over long periods of time, medication, or years of formal education (Ceci, 1991; Ceci & Williams, 1997; Deary et al., 2000). However, during the past decades, many researchers have questioned this view (e.g., Holmes et al., 2009; Jaeggi et al., 2011; Klingberg, 2010; Morrison & Chein, 2011). The interest in improving cognitive performance or in restoring deteriorated cognitive processes has led several laboratories to investigate the possibility of enhancing mental capacities by training the mind using different methods. One method that has received particular attention is computerized cognitive training (CCT). Several programmes have been designed, studied, and sold using this method, in which the user follows a regime of computer tasks during a period of time that ranges from a single session to several sessions across days or even months (e.g., Jaeggi et al., 2008; Klingberg et al., 2002; Olesen et al., 2004; Schmiedek et al., 2010; Shipstead et al., 2012). Performance of each of the tasks used in CCT programmes usually depends on a specific cognitive function; thus it is not unusual for programmes to include more than one computer training task.

The rationale behind cognitive training is that cognitive functions are malleable to a certain extent as a consequence of brain plasticity (Klingberg, 2010; Valkanova et al., 2014) and can therefore be improved if challenged appropriately. Neuroimaging studies have supported this view on several occasions. For example, a study investigating brain changes after a cognitive training programme (Chapman et al., 2015) found an increase of cerebral blood flow and greater connectivity in the executive network during resting-state. In another study investigating the effects of cognitive training in older adults using resting-state fMRI analyses (Cao et al., 2016), it was observed that, after training, participants who underwent a cognitive training programme showed better functional connectivity in the default mode network, salience network, and central executive network when compared with a control group who did not undergo the training programme. In a meta-analysis investigating neural changes after CCT, Li and others (2022) found increased activation in the left inferior frontal gyrus, an area commonly related to language and executive functions (Costafreda et al., 2006; Swick et al., 2008).

Clinical populations often exhibit unique characteristics that set them apart from healthy populations or even from other clinical conditions. For instance, attentional problems have been noted in individuals with ADHD (Slobodin et al., 2018) as well as in those with anxiety and depression (Armstrong & Olatunji, 2012). However, the nature of these attentional issues varies between clinical groups, suggesting that their specific rehabilitation needs are not identical. Consequently, a training programme effective for one population may not yield the same results for another group with differing characteristics. This further indicates that a programme designed to enhance cognitive functions in healthy adults may not be equally effective for addressing cognitive impairments in clinical populations. Thus, conclusions from studies focused on healthy individuals may not necessarily be applicable to clinical groups, leading to the development of two somewhat separate fields of research. The current review will concentrate solely on the impact of CCT on cognitive improvement in healthy adults.

## Working memory

Arguably, the cognitive function that has received the most attention in CCT is working memory (WM). WM is a complex cognitive system consisting of storage (short-term memory) and executive components that are responsible for maintaining and processing information (Baddeley, 2012). Studies have identified specific executive functions (EF) that regulate our thoughts and behaviour (Jewsbury et al., 2016; Miyake et al., 2000). Some of these include our capacity to inhibit prepotent or impulsive responses, switching attention between mental sets, and processing speed.

Since WM involves two components, experimental instruments used for its assessment are expected to tap both the storage and processing of information. For example, complex span tasks, a commonly used type of WM measure, require participants to store items in short-term memory (e.g., a list of words or numbers) while processing some other information (e.g., solving an equation or determining if a sentence makes logical sense). Simple span tasks, in which participants are required to recall a list of items in either forward or backward order, are also used to evaluate WM. However, some studies have shown that, in healthy young adult populations, performance of simple span tasks mostly depends on short-term memory and not on WM (Conway et al., 2002; St Clair-Thompson, 2010; St Clair-Thompson & Allen, 2013). Thus, simple span tasks may not be an appropriate measure of WM ability. One other task that has received significant attention, particularly in the neuroimaging field (Jacola et al., 2014; Owen et al., 2005), is the *n*-back task. In this task, participants are presented with a stream of stimuli and are asked to indicate each time the currently presented stimulus matches the stimulus presented *n* trials previously. The *n*-back task is commonly used as a measure of WM, and although task performance involves both storage and processing, some studies have suggested that it relies primarily on EF (Chatham et al., 2011; Kane et al., 2007). In addition to complex span and *n*-back tasks, updating tasks have also been used to assess the updating component of working memory. These tasks are very similar to *n*-back tasks. However, the key difference lies in the requirement for participants to recall the last ‘*n*’ stimuli only after the entire sequence of stimuli has been presented. This absence of responses during the presentation of stimuli might reduce the need for manipulation of these stimuli for successful performance. This is because participants are mainly required to update information in their short-term memory.

Many training programmes aim to enhance WM with the expectation that these gains will transfer to other cognitive skills, notably fluid intelligence. A cognitive ability highly related to academic and professional success (Schmidt & Hunter, 2004; Strenze, 2007) and that is understood to depend to some extent on WM. The notion of such benefits extending to unrelated cognitive processes is known as ‘far transfer.’ This is in contrast to ‘near transfer,’ where training effects apply to similar cognitive processes. Understanding the difference between far and near transfer is crucial for evaluating the impact of CCT on cognition, since enhancements in performance on trained or similar tasks may merely reflect practice effects, meaning that individuals are utilizing existing cognitive resources more efficiently rather than experiencing genuine cognitive improvement (Gobet & Sala, 2022).

## Computerized cognitive training

Two seminal studies by Klingberg et al. (2002) and Olesen et al. (2004) were among the first to evidence the trainability of WM and its effects on fluid intelligence. Their findings demonstrated significant improvements in both clinical and healthy populations after a brief regimen of multitask CCT consisting of several simple span tasks relying on working memory. Such groundbreaking results subsequently led to the establishment of a company offering cognitive training services, thereby underlining the potential utility and commercial viability of WM-focused training initiatives. Jaeggi et al. (2008) further contributed to this field, reporting consistent enhancements in intelligence among healthy young adults subsequent to single-task CCT training consisting of a dual *n*-back task. Such findings provided evidence that brief interventions like CCT can lead to significant cognitive improvements.

In both cases, the tasks incorporated in the training programs were tailored to adjust to each participant’s performance level. This personalization was achieved by modulating the quantity of stimuli the participant had to process based on their previous trial’s performance. By increasing the demands in both storage and processing—meaning recalling and dealing with more stimuli simultaneously—it was anticipated that participants would improve their short-term memory capacity and processing skills.

However, despite these promising results, attempts to reproduce these effects have posed challenges (Chacko et al., 2014; Smith et al., 2013). While boosting performance on a trained task in CCT studies is typically straightforward, demonstrating improvements in unrelated tasks has been more difficult. As the cognitive processes evaluated share fewer sub-processes with the trained task (e.g., attentional or inhibitory sub-processes), or when the assessment task diverges significantly from the training task (e.g., using a different stimulus type or varied paradigm), the observed benefits tend to be smaller or even absent (Melby-Lervåg et al., 2016).

It has been observed that a possible explanation for the lack of consistency in results between studies could be methodological weaknesses in their design (Gobet & Sala, 2022; Green & Newcombe, 2020; Green et al., 2019; Könen et al., 2016; Melby-Lervåg & Hulme, 2013; Shipstead et al., 2010). Some of the methodological criticisms relate to the absence of an adequate control group, problems regarding the instruments used for assessing transfer effects, small sample sizes, lack of adequate blinding of participants and researchers, how participants are assigned to groups (e.g., randomization), type of training used and availability of data.

Many studies have employed relatively small sample sizes (usually fewer than 50 participants; for reviews reporting sample sizes see Bogg & Lasecki, 2015; Hill et al., 2017; Kelly et al., 2014). These studies may therefore be underpowered, increasing the risk of making Type I (finding an intervention effective when it is not) and Type II (finding an intervention ineffective when it is effective) errors when interpreting the results. The lack of double-blinding in CCT studies has also been considered a limitation (Green et al., 2019), since it potentially increases the risk of bias introduced by the researcher (e.g., intentionally or unintentionally favouring certain results) and participants (e.g., placebo effects). Similarly, the procedures used for group assignment may represent a source of bias. In studies investigating the effects of an intervention, adequate randomization is commonly expected, however, Green et al. (2019) argue that in studies with small sample sizes, such as in CCT studies, randomization might produce unbalanced groups, leading to biased results.

The use of no-contact (passive) control groups—that is, participants who do not receive any form of intervention—has also been a matter of debate (Au et al., 2015; Boot et al., 2013; Melby-Lervåg et al., 2016; Melby-Lervåg & Hulme, 2013). One limitation with the use of passive control groups is that it cannot control for placebo effects and the occurrence of a phenomenon called the Hawthorne effect (McCambridge et al., 2014), in which participants behave differently as a consequence of being observed. It is possible that the participants’ performance may improve, not as a consequence of training a particular cognitive function, but rather due to increased effort during the posttraining assessment phase. One possible solution to this issue is to use an “active” control group—that is, a group undergoing a placebo training programme. This placebo programme is usually comparable to the experimental group in terms of length and schedule in order to exert the same expectation of improvement as is exerted by the experimental programme. The use of this type of control is grounded in the hypothesis that some or all of the transferable effects found as a consequence of CCT are derived from an increased effort in performing the posttraining assessment and not from a real improvement in cognitive functions. Most of the evidence supporting this hypothesis comes from larger effect sizes found when using passive control groups when compared with active control groups (Melby-Lervåg et al., 2016). Other meta-analyses, however, have not found significant differences between the posttraining scores of the active and passive control groups (Au et al., 2015, 2020).

## Meta-analyses in the field

In an attempt to provide a more definitive answer to whether or not CCT can deliver transferable effects, several meta-analyses have been performed. Unfortunately, as in the case of individual CCT studies, results are not sufficiently clear. For example, several meta-analyses have reported significant improvements of cognitive functions and transferable effects after training (Au et al., 2015; Cortese et al., 2015; Hill et al., 2017; Karbach & Verhaeghen, 2014; Kelly et al., 2014; Lampit et al., 2014; Leung et al., 2015). However, in a meta-analysis by Melby-Lervåg and Hulme (2016) in which data from two of these meta-analyses (i.e., Au et al., 2015; Karbach & Verhaeghen, 2014) were reanalyzed, it was argued that the significant results reported were obtained using biased methodologies. More specifically, Melby-Lervåg and Hulme observed biases in the selection of studies (e.g., not reporting what measures and effect sizes were coded, insufficient information about the selection of studies), calculation of effect sizes that did not consider baseline differences, and failures to consider possible placebo effects (by including studies using passive control groups). In another meta-analysis (Soveri et al., 2017), it was observed that the effect size of WM improvements reported by several meta-analyses was inflated by including untrained variants of the training task in the assessment of WM. Including such tasks in the assessment confounds improvements exclusively observed in the training task with real improvements in the construct of WM.

The multilevel meta-analysis by Soveri et al. (2017) and the meta-analysis by Melby-Lervåg et al. (2016) have provided important insights into the cognitive training field. For example, Melby-Lervåg et al. (2016) performed a close examination of near- and far-transfer effects of WM training programmes. This review presented several methodological strengths when compared with other reviews, such as including a larger number of studies (*k* = 87), comparing the effects of training when either an active or passive control group was used, and performing a specific analysis for criterion tasks—that is, assessment tasks that are very similar to those used for training. This latter methodological decision allowed the evaluation of practice effects, separating these effects from more valid transfer measures obtained using tasks less similar to those used during training. In this review, significant improvements were found in criterion tasks (near-transfer) and in other measures of WM (intermediate-transfer), however these improvements did not seem to translate into benefits for far transfer measures. In addition, the observed improvement in training groups versus active control groups was smaller than the improvement found in training groups versus passive control groups. Although the meta-analysis by Soveri et al. (2017) was limited to *n*-back training studies their results were similar: The effects of training may not translate into practical benefits.

The most common approach to meta-analytic investigation of the effects of cognitive training is to include only WM training programmes (i.e., Melby-Lervåg et al., 2016; Pappa et al., 2020; Schwaighofer et al., 2015; Soveri et al., 2017; Weicker et al., 2016)—that is, training programmes based on tasks primarily loading on WM, such as *n*-back, complex span, running span, or updating tasks. This has allowed studies to impute any far-transfer effect to improvements in WM. However, this also limits the scope of studies to be included. Examples of this include the study by Soveri et al. (2017), whose results are mostly limited to the *n*-back training programme and the study by Pappa et al. (2020), which only considered updating training programmes. Considering that WM is composed of a storage and executive component, it should be possible to improve WM by training either of these two components alone. For example, training EF could improve the executive component of WM, allowing it to process information more effectively. We consider only including WM training programmes to be a limitation of previous meta-analyses since WM might be improved by training either of its two components. The quality of CCT studies is also commonly discussed in many meta-analyses since it has been found that certain characteristics of the studies, such as the sample size or the use of active control groups, have an impact on the effect of training (Melby-Lervåg et al., 2016; Melby-Lervåg & Hulme, 2016; Sala & Gobet, 2017, 2020; Soveri et al., 2017). However, there are other methodological decisions in each study that could represent a source of bias in the reported results, such as the method used for assigning participants to groups or selective reporting of instruments and analyses. To date, no meta-analysis evaluating the effects of cognitive training on WM of healthy adults has performed a risk of bias assessment of their included studies and included programmes training other processes different than WM.

## The present study

The current review has two primary objectives: to evaluate the impact of CCT on specific cognitive functions (mainly WM) and to examine the influence of methodological concerns raised in prior studies on the reported effects. To accomplish these aims, we will explore the following specific research questions:

1. Is it possible to improve WM by following computerized cognitive training programmes?
2. To what extent do practice effects contribute to reported effects of CCT?
3. Does enhancing working memory lead to an increase in fluid intelligence?
4. Do CCT interventions also influence executive functions and short-term memory?
5. What is the likelihood of encountering biased results due to questionable methodological practices in the field of CCT?
6. To what extent do methodological choices, such as the use of active versus passive control groups, training intensity, compensation, sample size, and type of training, influence the reported outcomes?

These research questions will be explored through a systematic review of CCT programmes that have assessed WM. This includes not just those specifically designed for WM training, but any CCT programme that evaluates effects on WM. Although the effects of CCT on cognition have been studied on numerous occasions, we provide an updated perspective and a closer examination of most of the concerns raised in the field over the years.

The first two questions explore the effects of CCT on working memory. A meta-analysis was conducted to estimate the average effect of training on WM performance. To control for practice effects, only studies employing different tasks for training and assessment were included, and a secondary analysis examined results from studies using an assessment task that was very similar to the training task. Furthermore, to assess genuine improvement in WM, tasks selected for assessment were required to involve both the storage and processing of information. Simple span tasks, which rely primarily on short-term memory, were not considered suitable WM assessment measures in this review. The third question evaluates whether CCT can enhance fluid intelligence and whether these gains are attributable to improvements in working memory. The relationship between working memory improvements and changes in fluid intelligence was examined through a meta-regression with WM as a moderator.

In the fourth question, we evaluate whether CCT can improve other cognitive processes, such as specific executive functions and short-term memory, through an additional set of meta-analyses. To address the fifth question, a Risk of Bias assessment was conducted on each study included in the review. An additional analysis was then performed, specifically omitting studies deemed to have a high risk of bias, to identify any discrepancies in outcomes. Finally, for the last question, separate analyses were conducted for passive control groups, active control groups, and a pooled analysis of both to examine differences in effect size. To investigate the effects of other methodological choices, a series of meta-regressions were employed to evaluate whether training intensity, sample size, compensation and type of training moderate the effects of training on WM.

Considering that different populations (children, older adults or people with a clinical condition) may present very specific characteristics that can potentially moderate the effects of training, we opted to investigate the effects of training in healthy young and middle-aged adults only. In this sense, the present study represents an investigation of cognitive enhancement and not of cognitive rehabilitation.

In summary, this meta-analysis aims to address persistent questions in the field that have accumulated over years of research, necessitating a more direct approach. Our goal is to provide an updated review of the evidence concerning the effects of working memory, while also tackling prevalent methodological issues that have hindered researchers from drawing confident conclusions. Despite important contributions from several meta-analyses, new methodological challenges continue to be identified. These include factors not previously examined collectively, such as participant allocation procedures, study pre-registration, the influence of practice effects in meta-analytic results, and the nature of control measures used to assess training effects. Furthermore, we assess the impact of CCT on EFs and explore how specific study characteristics might enhance training outcomes.

## Method

### Search and selection of studies

**Fig. 1:**
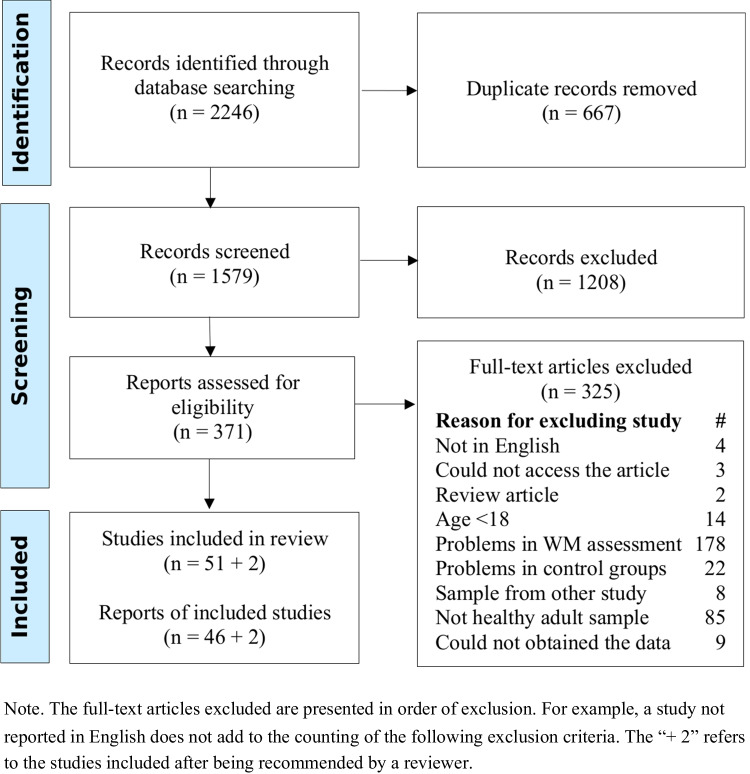
Process of study selection

This process is represented in Fig. 1. The literature search was performed using the PsycINFO and MEDLINE (accessed through PubMed) databases on 21 June 2019 and updated on 25 August 2022 and 22 May 2023. The search strings required the term “working memory” to be present anywhere in the article, in addition to the term “cognitive training” or “working memory training” or “brain training” or “memory training” or “computer training” or “computerised training”. The final search returned 1,177 articles from PsycINFO and 1,100 from PubMed. Duplicate articles were removed using Mendeley Desktop (https://www.mendeley.com/autoupdates/installers/1.19.5) and Covidence systematic review software (www.covidence.org) resulting in a total of 1,580 articles for title and abstract screening. The web-application abstrackr (Wallace et al., 2012) was used for the screening of titles and abstracts conducted in 2019 and Covidence systematic review software for the screening conducted in 2022 and 2023.

The inclusion and exclusion criteria for this study were (a) the training programme must have been delivered through a computer, (b) participants must be young or middle-aged adults (between 18 and 64 years of age) without a psychological disorder, (c) the design of the study must have been experimental or quasi-experimental, (d) either an active or a passive control group must have been used, (e) WM must have been measured before and after training, (f) at least one objective measure of WM must have been used, (g) at least one of the objective instruments used for assessing WM must have been different from those used for training, (h) the tasks used for assessing WM must require both storage and processing of information (e.g., *n*-back task, complex span tasks, or updating tasks), (i) studies must have been reported in English to avoid misinterpretations due to difficulties with translation. Tasks primarily relying on short-term memory with very low processing requirements, such as simple span tasks, were not considered as WM measures in young and middle-aged adults. While our focus was on studies involving young and middle-aged adults, we did include studies that also featured a small number of older adults due to their age-range criteria (e.g., adults between 40 and 70 years of age).

The screening process was conducted by JAR and CMG and is depicted in Fig. 1 following the PRISMA Flow Diagram (Page et al., 2021). The title and abstract screening was performed by both authors, and a near-perfect agreement was achieved (K = 0.92). For the full-text screening, JAR screened all articles and CMG a randomly-selected 25%. Again, near-perfect agreement was obtained (K = 0.91).

In addition to the studies identified from this search, two additional studies were included after being recommended by a reviewer. Both studies fulfilled the inclusion criteria.

### Risk of bias assessment and coding

After the screening was completed, each study underwent a risk of bias assessment using the RoB 2 tool (Sterne et al., 2019) and data were extracted into a database. The RoB 2 is an updated revision of the Cochrane Risk of Bias Tool, one of the most commonly-used tools for assessing the validity of the results of randomized controlled trials. This instrument evaluates the risk of bias from five different sources (domains): (1) from the randomization process, (2) due to deviations from intended interventions, (3) due to missing outcome data, (4) in measurement of the outcome, and (5) in the selection of the reported results. The tool provides a set of questions covering key aspects of each domain. Each question aims to identify specific features of the study that are related to sources of bias. Depending on the answers provided a judgement is made regarding the risk of bias within each domain and at the study level. In addition to assessing the risk of bias, we evaluated publication bias using a funnel plot and examined its asymmetry through a regression test. This approach enables us to assess potential biases arising from small studies and file drawer effects, which refer to the practice of not publishing studies with small sample sizes and nonsignificant results.

WM measures were categorized either as verbal or nonverbal depending on the type of memory required for the task. Tasks in which the elements to be remembered or processed could be expressed verbally (e.g., numbers, letters, directions, etc.) were classified as verbal WM. In all other cases, the tasks were classified as nonverbal WM. Some studies also included tasks very similar in structure and type of stimuli to those used for training. These tasks were coded as criterion WM tasks and analyzed separately. When more than one paper reported data from the same sample and using the same instruments, only the study presenting the most complete data was included. This was done to avoid using the same sample twice in the same comparison (independence of observations). In some instances, an article featured multiple studies (Jaeggi et al., 2008; Pahor et al., 2022; Sprenger et al., 2013) or contained more than one experimental group along with a corresponding control group (Chooi & Thompson, 2012). In such cases, each comparison was treated as a separate entity and included individually in our analysis. It was common to find studies using more than one instrument for measuring the same type of WM. In these cases, the mean was calculated for all tasks of the same type (verbal, nonverbal or criterion). A similar procedure has been used in prior meta-analyses (e.g., Melby-Lervåg et al., 2016; Melby-Lervåg & Hulme, 2013; Schwaighofer et al., 2015). For the present study, only pre- and posttraining measures were used. Measures obtained during training or follow-up assessments were not considered.

In addition to WM, data from assessments of other cognitive functions were also extracted, including measures of fluid intelligence, short-term memory, processing speed, inhibition and attention switching. Due to the relatively small number of studies reporting these functions, we did not distinguish between the type of stimuli (e.g., verbal or nonverbal) used in any of these measures. Only data from experimental tasks were considered. Again, when more than one task was used for assessing a particular function, a weighted average was calculated as the outcome score by taking into account the number of observations (n) for each of the scores included in the average. For this, we calculated the weighted sum of each task score by multiplying it by the number of participants who completed that task. We then added these weighted sums together. Additionally, we calculated the total number of participants across all tasks. Finally, we divided the aggregated weighted sum by this total number of participants.

The type of training used was coded either as (a) single task, when only one task heavily relying on one cognitive process was used for training (e.g., training WM with a complex span task); (b) multitask, when more than one task relying on the same process was used for training (e.g., training WM using complex span tasks and *n*-back tasks); and (c) multiprocess when more than one cognitive function was trained using different tasks (e.g., training attention and WM using several tasks). We also coded whether the training was carried out in the laboratory or at each participant’s home. Other moderator variables include dosage in number of minutes dedicated to training, total economic compensation in US dollars, and size of training groups.

### Analyses

Three types of analysis were performed in this study: A risk of bias analysis, meta-analyses, and meta-regressions. Heterogeneity was measured using *I*^2^, which presents the variability between effect estimates not explained by chance and is presented in the form of a percentage (Higgins, 2003).

Following recommendations from Morris (2008), we computed the standardized mean difference (SMD) using the difference between pre- and posttraining assessments and the pooled standard deviation from the pretraining scores only. Pretraining scores were subtracted from posttraining scores, and control group scores were subtracted from experimental group scores. This procedure ensured that positive values represented an improvement and negative values a decline in performance when compared with a control group. Meta-analyses were performed in R (R Core Team, 2021) with the “metafor” package, using a random-effect model with the restricted maximum likelihood method. Meta-regressions were performed to determine the effects of categorical and continuous moderator variables on training effects. This procedure allows the implementation of linear regression principles in meta-analyses to calculate the impact of moderator variables (Huizenga et al., 2011) and to explain the heterogeneity between studies (Baker et al., 2009). Since the third question of this review asked whether improvements in WM could transfer to fluid intelligence, we performed a meta-regression to determine the proportion of these effects that could be attributed to variations in WM. For this meta-regression, we used the effect sizes obtained from the meta-analysis evaluating the effects of cognitive training on WM as the mediator variable.

Some studies included in the review compared different CCT programmes to the same control group. In these cases, training groups that fulfilled the inclusion criteria were pooled together into a single group since creating two comparisons since using the same control group would have violated the assumption of independence of effects. In cases where more than one control group was used (i.e., passive and active), the two control groups were pooled together. Main analyses were performed using these pooled groups whenever available. Additional analyses were performed using only active and only passive control groups. We also performed separate analyses for the different types of WM measures (i.e., verbal and nonverbal) and the criterion tasks.

## Results

For the presentation of our results, we will first provide an overview of the included studies. This will be followed by a qualitative evaluation of the risk of bias. We will then present results from meta-analyses examining WM under different conditions, including a comparison of active versus passive control tasks, an examination of studies using assessment tasks very similar to the training task and an analysis excluding studies with a high risk of bias. Next, we will evaluate effects of CCT on fluid intelligence, executive functions, and short-term memory. Finally, the outcomes of the meta-regressions evaluating the impact of various methodological choices on training effects will be presented. In the discussion section, we will address our research questions in light of these findings.

### Description of studies

Details of the included studies, such as type of training, size of experimental groups, dosage of training and compensation, can be found in Table 1. Several articles provided more than one comparison by reporting more than one study or by using training groups with a corresponding control group. In all these cases, we assigned a different identifier for each comparison.

**Table 1:** Description of included studies

ID	Design	Training place	Mean age	Age *SD*	Type of training	Target process	# sessions	Total minutes	Compensation	Experimental group size	Training tasks
Baniqued et al., 2014	RCT	Laboratory	21	2.3	Multiprocess	WM, reasoning, EF	10	800	200	40	4 games involving planning, abstract reasoning and WM
Baniqued et al., 2015	RCT	Laboratory	21.01	2.2	Multiprocess	STM, WM, reasoning	20	240	60	39	6 tasks: STM, WM, reasoning
Blacker et al., 2017	RCT	Home	21.18	3.12	Single task	WM	20	600	215	136	Dual *n*-back (audio and visuospatial) / Automated symmetry span
Chooi and Thompson, 2012	RCT	Laboratory	20	NR	Single task	WM	8	240	0	15	Dual *n*-back task
Chooi and Thompson, 2012	RCT	Laboratory	20	NR	Single task	WM	20	600	30	16	Dual *n-*back task
Colom et al., 2013	NonRCT	Laboratory	18.1	1.1	Single task	WM	24	720	266	28	Dual *n*-back task
Desmarais and Vachon, 2023	RCT	Laboratory	23.91	5.06	Multiprocess	WM, EF	8	240	60	40	*N*-back task, response competition
Fellman et al., 2020	RCT	Home	34.41	8.45	Single task	WM	12	360	70	191	*N*-back task
Flegal et al., 2019	NonRCT	Home	20.8	2.4	Multitask	WM	10	600	200	19	Matrix updating and Keep track: both required STM and some WM processing
Foster et al., 2017	RCT	Laboratory	22.2	NR	Multitask	WM	20	950	300	39	Verbal and spatial complex span tasks / Verbal and spatial running span tasks
Hayashi et al., 2016	RCT	NR	24.4	1.5	Multiprocess	STM, EF	25	875	Gift	31	CogMed: simple span tasks
Henshaw et al., 2021	RCT	Home	64.98	5.88	Multitask	WM	25	1000	46	26	CogMed: simple span tasks
Hofmann and Förster, 2019	RCT	Home	24.1	4.5	Multiprocess	WM, EF	18	NR	50	52	OSPAN, *N*-back, colour-shape task, number-letter task, stop-signal task, Stroop task
Holmes et al., 2019	RCT	Home	28.8	6.9	Multitask	WM	20	400	40	16	Verbal *n*-back, visuospatial *n*-back / Verbal complex span, visuospatial complex span
Jaeggi et al., 2008	NonRCT	Laboratory	25.6	3.3	Single task	WM	12	300	NR	11	Dual *n-*back task
Jaeggi et al., 2008	NonRCT	Laboratory	25.6	3.3	Single task	WM	17	425	NR	8	Dual *n*-back task
Jaeggi et al., 2008	NonRCT	Laboratory	25.6	3.3	Single task	WM	19	475	NR	7	Dual *n*-back task
Jia et al., 2023	RCT	N/A	50.3	11	Multitask	WM	23	805	NR	58	CogMed: simple span tasks
Kattner 2021	RCT	Laboratory	24.80	7.1	Single task	WM, EF	8	720	0	23	Dual *n*-back task, inhibitory *n*-back and N/A
Kundu et al., 2013	RCT	Laboratory	20.9	2.75	Single task	WM	25	1250	NR	13	Dual *n*-back task
Li et al., 2022	RCT	Laboratory	21.9	2.15	Multitask	WM	40	1200	NR	40	Simple span tasks
Linares et al., 2019	RCT	Laboratory	21.4	6.2	Multitask	WM	6	180	Credits	63	Arithmetical updating task, number size updating task / Letter *n*-back task, number *n*-back task
Luo et al., 2017	RCT	Laboratory	21	1.5	Single task	WM	15	375	30	16	Spatial *n*-back task
Maraver et al., 2016	RCT	Laboratory	20.5	1.73	Multitask	WM, EF	6	480	NR	28	Three games, all requiring STM, conflict resolution, switching / *N*-back; WM search, category updating
Matzen et al., 2016	RCT	Laboratory	37	NR	Multitask	WM	15	375	NR	25	Dual *n*-back task
Minear et al., 2016	RCT	N/A	19.8	1.5	Single task	WM	20	400	300	29	Visuospatial *n*-back / Verbal complex span
Nouchi et al., 2013	RCT	Home	20.7	1.2	Multiprocess	Crystallised skills	20	300	NR	16	Brain Age (Nintendo Co. Ltd.): reading, maths, memory.
Nouchi et al., 2020	RCT	Home	20.53	0.27	Multiprocess	Crystallised skills	28	560	NR	36	Brain Age (Nintendo): arithmetics calculation, reading, syllable count, select number from lower to highest, count number of people, calculations
Oelhafen et al., 2013	RCT	Home	25.2	4.1	Single task	WM	14	350	50	15	Dual *n*-back
Pahor et al., 2021	RCT	NR	20.47	4.41	Single task	WM	13	400	NR	49	*N*-back task
Pahor et al., 2022	RCT	Laboratory	20.26	1.74	Single task	WM	20	400	120	72	*N*-back task
Pahor et al., 2022	RCT	Laboratory	20.29	2.63	Single task	WM	20	400	120	109	N-back task
Pahor et al., 2022	RCT	Home	20.6	2.95	Single task	WM	20	400	80	128	*N*-back task
Parong et al., 2022	RCT	Home	20.18	13.37	Single task	WM	20	400	170	62	*N*-back task
Penner et al., 2012	RCT	Home	39.3	2.6	Multiprocess	WM, STM, reasoning	16	720	0	12	BrainStim: spatial orientation, visual STM, digit complex span
Redick et al., 2013	RCT	Laboratory	21	2.6	Single task	WM	20	600	40	24	Dual *n*-back
Redick et al., 2019	RCT	Laboratory	20.5	2	Single task	WM	10	300	145	29	Operation-letter span / Operation-mix span
Roberts et al., 2021	RCT	Home	19.54	2.05	Single task	WM	20	460	NR	26	Manipulation of items within STM
Rodas and Greene, 2021	RCT	Home	22.85	7.60	Single task	WM	20	500	0	42	Single *n*-back task
Salmi et al., 2019	RCT	Laboratory	21.9	2.99	Single task	WM	15	338	112	20	Dual *n*-back task
Salminen et al., 2012	RCT	Laboratory	24.5	3.7	Single task	WM	16	480	71	17	Dual *n*-back task
Salminen et al., 2016	RCT	Laboratory	24.5	NR	Single task	WM	14	420	72	20	Dual *n*-back task
Schmiedek et al., 2010	RCT	Laboratory	25.6	2.7	Multiprocess	EF, STM, WM	100	7500	1700	73	12 tasks: processing speed, episodic memory, WM
Scholl et al., 2021	RCT	Home	22.06	1.99	Multitask	WM	17	420	0	29	Neuronation: 10 WM tasks
Shahar and Meiran, 2015	RCT	Laboratory	25.1	1.6	Multitask	EF	19	1140	114	16	Object classification task and spatial classification task
Shahar et al., 2018	RCT	Laboratory	23.72	NR	Single task	EF	14	420	NR	72	Focus switching task combined with an *n*-back procedure.
Soveri et al., 2017	RCT	NR	20.1	2.2	Single task	WM	15	375	79	25	Dual *n*-back task
Sprenger et al., 2013	RCT	Laboratory	23	NR	Multiprocess	WM, STM, EF	20	1200	500	57	Letter *n*-back, auditory running span, block span, letter-number sequencing, and tasks developed by Posit Science (match-it, sound-replay, listen-and-do, and jewel-diver)
Sprenger et al., 2013	RCT	Home	35.51	9.14	Multiprocess	WM, EF, STM	30	780	100	35	*N*-back task and a task combining flanker and Stroop tasks / 2 block span tasks
Thompson et al., 2013	RTC	Laboratory	21.87	NR	Single task	WM	20	800	20	20	*N*-back task
von Bastian et al., 2013	RCT	Home	23	4	Multiprocess	WM, EF	20	600	127	33	Complex span tasks (verbal, numerical, and figural-spatial) / Relational integration tasks (letter, kinship, pattern) / Task switching (pictorial, verbal, figural)
von Bastian and Oberauer, 2013	RCT	Home	23.3	3.9	Multitask	WM, EF	20	600	160	30	Numerical complex span, Tower of Fame (follow instructions, required WM), figural task switching
von Bastian and Eschen, 2016	RCT	Home	23	3.01	Multitask	WM	20	800	88	34	Three complex span tasks: numerical, verbal, and figural-spatial
Ward et al., 2017	RCT	Laboratory	25.4	NR	Multiprocess	WM, reasoning, EF	48	3360	NR	63	Dual *n*-back, reasoning, switching, planning, visuospatial WM, updating
Zhao et al., 2023	RCT	Laboratory	19.75	1.5	Single task	WM	20	600	16.5	33	*N*-back task

Different programmes on the same study are separated by a slash (“/”)

# sessions = Total number of sessions according to the mean number of sessions reported by authors; Time in minutes = Mean total minutes dedicated to training; Compensation = Mean total compensation in USD given to participants (other currencies were converted to USD according to the mean exchange rate of the year of publication of the study); Experimental group size = Number of participants on experimental groups; RCT = Randomized controlled trial; NonRCT = Nonrandomized controlled trial; Multiprocess = Training aiming at multiple cognitive functions; Multitask = Training using multiple tasks to train a single cognitive process; Single task = Only one task used for training; WM = Working memory; EF = Executive functions; STM = Short-term memory; NR = Information not reported

All studies included in this review were published between 2008 and 2023 in peer-reviewed journals. From the studies included in this review, 48 were randomized control trials, two studies had a nonrandomized parallel-group design, and three studies did not report how the sample was distributed between groups. The mean age of participants from all the included studies was 24.6 (*SD* = 3.73), and the mean size of experimental groups was 40.92 (*SD* = 34.59), ranging from 7 to 191 participants. The number of training sessions ranged from 6 to 48, except for one study that reported 100 sessions (Schmiedek et al., 2010). The mean number of sessions was 20.09 (*SD* = 13.42), and 18.56 (*SD* = 7.48) when excluding the study with 100 sessions. Thirty-five studies reported paying participants as a form of compensation (*M* = $151.58 USD, *SD* = $282.26).

### Risk of bias in included studies

While Domains 1, 2, 3, and 5 from the RoB 2 involve general characteristics of the study, Domain 4 is specific to the instrument used for the analyses, usually requiring one individual risk of bias assessment per instrument. However, due to the inclusion criteria, all WM instruments shared important characteristics such as being experimental tasks, objective measures, and in common use for the assessment of WM. This made individual assessments of each task unnecessary, leading us to perform only one risk of bias assessment for all included tasks within each study.

**Fig. 2:**
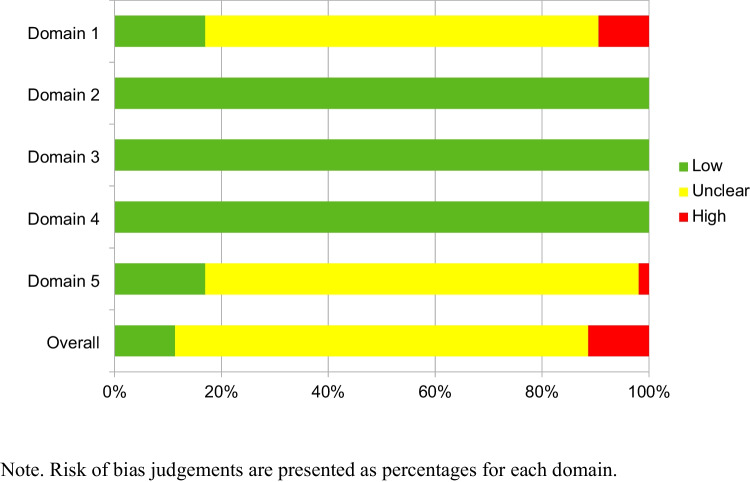
Risk of bias across studies grouped by domain

Since the RoB 2 was designed to assess randomized controlled trials, it assumes that an intervention group is being compared with a specific control group. In studies where both an active and a passive control group were used, we only considered the active control group for the assessment. Figure 2 represents the authors’ judgement about each domain as accumulated percentages. Further details of this assessment can be found in the Supplemental Materials S1 presents details on the Risk of Bias assessment, and S2 summarizes the risk of bias presented by each study in each Domain.

In general, the included studies present a very homogeneous risk of bias with only the randomization process (Domain 1) and selection of reported results (Domain 5) presenting possible sources of bias. A common limitation observed was a lack of reporting about how the random sequence for distributing the sample across groups was generated. The other common limitation was the lack of pre-registration of the instruments and analysis used, which leaves open the possibility that these studies selected which outcomes and analyses to report after the data were analyzed. Very few studies preregistered the outcome measures and analysis plan (i.e., Fellman et al., 2020; Henshaw et al., 2021; Pahor et al., 2022; Rodas & Greene, 2021). In one case (Henshaw et al., 2021), the outcome measures and analysis plan were pre-registered, however, different analyses were reported to those planned. In relation to bias derived from deviations from the intended interventions (Domain 2), due to missing outcome data (Domain 3), and in measurement of the outcome (Domain 4), all studies presented very similar characteristics with low risk of bias. For example, there were no reports of participants receiving CCT treatments other than the one assigned (Domain 2), it was very unlikely that missing outcome data depended on its true value (e.g., attrition rate did not seem related to training programmes; Domain 3), and none of the instruments used could be influenced by knowledge of the intervention received, since the instruments used registered objective responses, such as response times or correct answers (Domain 4). Six studies presented an overall high risk of bias (i.e., Colom et al., 2013; Flegal et al., 2019; Henshaw et al., 2021; all studies included in Jaeggi et al., 2008). In five of these cases, it was due to the lack of randomization of the sample.

**Fig. 3:**
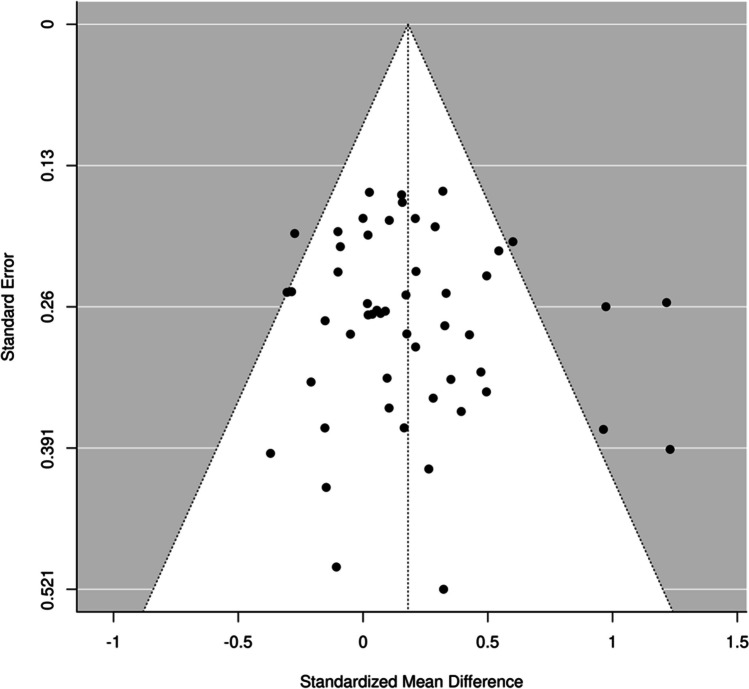
Funnel plot

Publication bias was evaluated using a funnel plot (Fig. 3). As observed in the figure, no signs of asymmetry can be seen, and a regression test further corroborates this (*t* = 0.828, *df* = 50,* p* = 0.412). Thus, our results are unlikely to have been affected by file-drawer problems or other forms of publication bias.

### Effects of cognitive training on working memory

**Fig. 4:**
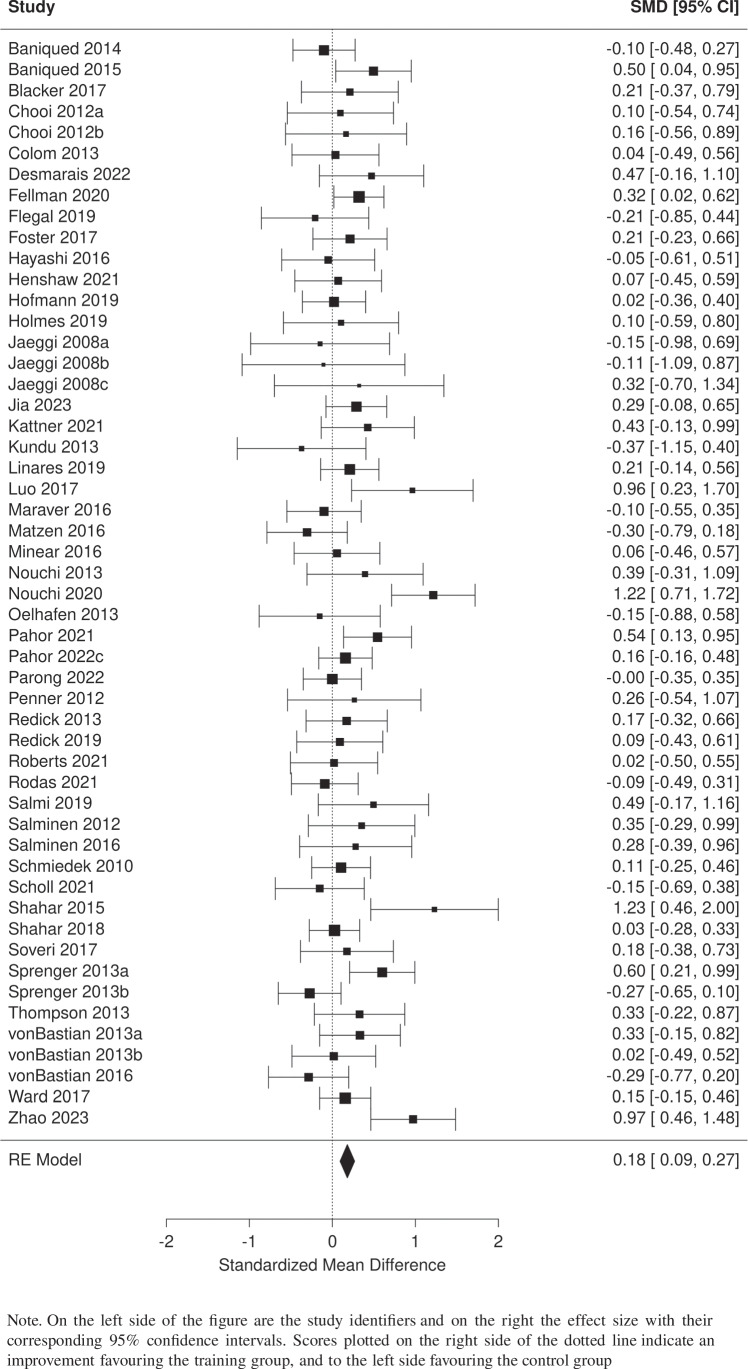
Forest plot of the effects of computerized cognitive training on working memory from pooled training groups vs pooled control groups

We first analyzed the main effect of training on WM by comparing the experimental groups versus the pooled control groups using verbal and nonverbal WM tasks (excluding criterion tasks—i.e., tasks that were very similar to the training task). This analysis consisted of 52 independent comparisons including 3,737 participants. Significant heterogeneity was found between studies (*I*^2^ = 38%). Results indicate a small but significant aggregate effect of training on WM (SMD = 0.18, 95% CI [0.093, 0.268], *p* < .001). The forest plot presented in Fig. 4 details the results of this analysis.

Table 2 summarizes the results from six different meta-analyses. The first two compared training groups versus (1) active control, and (2) passive control groups. In both cases, results were significant and very similar in effect size to the results obtained from combining both groups (Fig. 3). The next set of analyses were focused on the type of WM assessed. For these, active and passive control groups were pooled into a single group in cases where both types were used in the same study. Training groups were compared with control groups on (3) verbal WM, (4) nonverbal WM, and (5) criterion tasks. Significant effects of training were observed in all cases; however, the effect size observed in the criterion tasks was by far the largest. One additional analysis (6) was performed excluding studies from the main analysis that presented a high risk of bias. As observed from the table, results from this analysis remained significant and were very similar to the results found when all studies were included.

**Table 2:** Results from meta-analyses on WM using specific groups and tasks

		Comparisons	Participants	Heterogeneity	SMD (*p*)	95% CI
1.	Active control^a^	35	2,489	60%	0.19 (0.004)	0.061 to 0.323
2.	Passive control^a^	26	1,494	12%	0.19 (0.001)	0.072 to 0.297
3.	Verbal WM^b^	47	3,350	53%	0.18 (0.001)	0.068 to 0.282
4.	Nonverbal WM^b^	24	2,037	0%	0.15 (0.001)	0.063 to 0.242
5.	Criterion tasks^b^	18	1,521	95%	1.14 (<.001)	0.642 to 1.633
6.	Excluding studies with high risk of bias^c^	44	3,461	45%	0.19 (<.001)	0.097 to 0.291

^a^Pooled training groups versus one type of control group including verbal and nonverbal WM tasks

^b^Pooled training groups versus pooled control groups including one type of WM task

^c^Pooled training groups versus pooled control groups including verbal and nonverbal WM tasks

### Effects of cognitive training on fluid intelligence

**Fig. 5:**
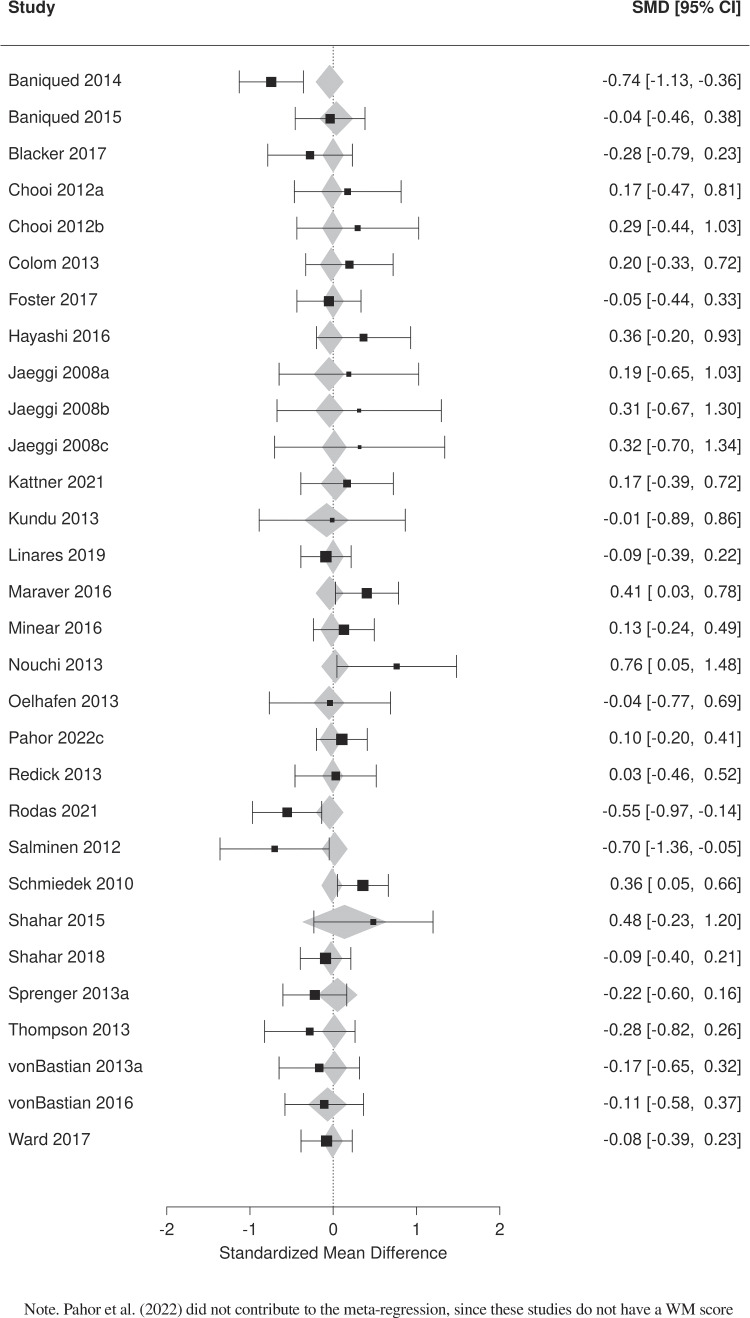
Forest plot of effects of computerized cognitive training on fluid intelligence with WM effect size as a moderator variable

A meta-analysis evaluating the effects of cognitive training on fluid intelligence was performed using pooled training groups versus pooled control groups. The analysis involved 33 independent comparisons including 2,729 participants. Significant heterogeneity was found (*I*^2^ = 44%) and the aggregate effect was not significant (SMD = 0.007, *p* = .908, 95% CI [−0.105, 0.118]). We investigated the possibility that the magnitude of the effect of CCT on fluid intelligence may be related to the magnitude of the WM training effect. To evaluate this, a meta-regression was performed using the main WM effect sizes (pooled training vs. pooled control) as a moderator variable. This analysis did not show a significant effect of the moderator variable in the model (coefficient = 0.135, *SE* = 0.228, *I*^2^ = 47%, *p* = .556), indicating that the effects of training on WM do not moderate the effects on fluid intelligence. Figure 5 presents the results from these analyses in a forest plot, which includes both the SMD of each study as a black square and the fitted value as a grey polygon.

### Effects of cognitive training on executive functions and short-term memory

Many of the included studies investigated the effects of training on EF and short-term memory. Again, for these analyses we compared the pooled training groups with pooled control groups. As observed from Table 3, significant effects of training were found on inhibition. In the other cognitive processes no effects were found and high levels of heterogeneity were observed. An inspection of the forest plot from these analyses (presented in the supplementary materials, Fig. S3 and S4) reveals that this heterogeneity might be due to a single study (Jia et al., 2023). In a sensitivity analysis, removing this study resulted in a decrease in heterogeneity and a significant effect of training in both processing speed (k = 12, *I*^2^ = 22, SMD = 0.192, *p* = .036) and short-term memory (k = 26, *I*^2^ = 2, SMD = 0.146, *p* = .003).

**Table 3:** Results from meta-analyses of executive functions and short-term memory

	Comparisons	Participants	Heterogeneity	SMD (*p*)	95% CI
Inhibition	24	1,801	24%	0.19 (<.001)	0.080 to 0.296
Switching	16	1,259	84%	−0.05 (.701)	−0.325 to 0.218
Processing speed	13	721	93%	−0.1 (.767)	−0.675 to 0.498
Short-term memory	27	1,775	80%	0.05 (.632)	−0.164 to 0.270

### Effects of moderator variables on WM

We also evaluated whether various characteristics of the studies moderated improvements in WM. For this, a series of meta-regressions were performed in which the effect size from the training intervention was the outcome variable. The moderator variables analyzed were the total minutes dedicated to training, total economic compensation, training group size, training place (laboratory or home), and training type—that is, whether the programme trained using a single task targeting a specific process (single-task; used as the reference group in the analysis), multiple tasks targeting a single process (multitask), or multiple tasks targeting multiple processes (multiprocess). Each moderator was analyzed independently—that is, not as a single model. None of these variables was found to be a significant moderator of the effects of training. Results from these analyses can be found in Table 4.

**Table 4:** Results from meta-regressions on the effects of WM

Moderator	Regression coefficient (*SE*)	95% Confidence Interval			
		Lower	Upper	*p*	*I*^2^ %
Total minutes	0^a^ (0^a^)	0^a^	0^a^	0.813	37
Total compensation	0^a^ (0^a^)	0^a^	0^a^	0.869	17
Training group size	0^a^ (0.002)	−0.002	0.004	0.581	35
Training place (Lab)	0.09 (0.099)	−0.106	0.283	0.374	37
Training target (Multitask)	−0.08 (0.11)	−0.133	0.3	0.448	36
Training target (Multiprocess)	0.06 (0.116)	−0.312	0.141	0.46	36

^a^Values are equal or lower than 0.001

*SE* = standard error

Each moderator was analyzed independently. Home and single tasks were used as the reference level of the variables training place and training target, respectively

## Discussion

In this review, we aimed to offer a comprehensive and updated meta-analysis of the effects of CCT on several cognitive functions, namely working memory, specific executive functions, and short-term memory. Furthermore, we assessed the extent to which different sources of bias may impact reported outcomes in the field. In general, we found small but significant improvements in multiple cognitive processes, which could be attributed to CCT. We examined the potential for biased results from several angles, including an assessment of methodological choices and publication bias. Although several common limitations were observed in the included studies, our findings suggest that the most impactful source of bias was the inclusion of practice effects in the WM outcome measures, which frequently results in an overestimation of training effects.

Our first two questions examined whether it is possible to improve WM using CCT and to what extent practice effects contribute to reported effects of CCT. According to our results, CCT can lead to significant improvements in WM, although the effect is very small (SMD = 0.18). This could explain the lack of transferable effects to other complex cognitive processes and skills. For example, Melby-Lervåg et al. (2016) evaluated far-transfer effects of WM training on complex skills such as reading comprehension, arithmetic, and verbal abilities and found no evidence of improvements, although significant improvements in WM were found after training. It is important to consider that performance of complex skills such as those investigated by Melby-Lervåg et al. depend on multiple factors of which WM is only one (Daneman, 1991; Peng et al., 2016, 2018; Raghubar et al., 2010). Thus, small improvements in WM may be insufficient to produce improvement in those skills. Results from meta-analyses of both verbal and nonverbal WM tasks were significant and very similar in strength. This seems to indicate that training does not have a differentiated effect on different types of WM. The second question was addressed by conducting an analysis including only tasks similar to the training task, which were excluded from the primary analyses. In contrast to the results found in the main analysis, these effects were very large, indicating that practice effects are strong and may appear on untrained but similar tasks (e.g., those sharing the same paradigm). This result highlights the importance of excluding criterion tasks from analyses since the training effect size can be distorted by practice effects. If prior studies did not take sufficient care in controlling for criterion tasks in the assessment scores included, their estimates could be inflated by practice effects giving a false impression of larger effect sizes. This also raises the question of how similar the task must be to be affected by training, and which are the paths of transfer. We opted to separate tasks based on their paradigm, since tasks from a similar paradigm (e.g., Operation Span Task, Symmetry Span Task, Reading Span Task) may allow for similar strategies to be used. Although this approach resulted in stricter inclusion criteria (fewer studies included), it allowed us to better control for practice effects. Although some research exists on the paths of transfer (Harris et al., 2020; Sprenger et al., 2013), more emphasis should be given to this area.

For the third question, we analyzed if CCT could induce improvements in fluid intelligence, a complex cognitive ability that some studies have shown to be improved with *n*-back training programmes (Au et al., 2015; Soveri et al., 2017). It is worth noting that four of the included studies specifically trained fluid intelligence, while the rest trained a variety of cognitive functions predicted to affect intelligence. Despite this, we did not find any significant effect. Results from a meta-regression using posttraining changes in WM as a moderator variable showed that the effects of training on WM are not related to the effects on fluid intelligence*.* However, the improvements found in WM are very small, and WM accounts for only part of the variance in fluid intelligence (Kane et al., 2005). Thus, if improvements in fluid intelligence do depend on improvements in WM, a relatively large change in WM ability may be required to produce a noticeable effect on fluid intelligence.

Our fourth research question asked whether it is possible to improve EF and short-term memory, since these are core functions for planning and directing behaviour. We found evidence of improvements in inhibition, processing speed and short-term memory. Prior studies investigating healthy adults have also reported improvements in EF (Nguyen et al., 2019; Soveri et al., 2017). However, these tend to evaluate EF very broadly, incorporating very different processes within this category (Webb et al., 2018). Although complex behaviour requires all EF to operate in conjunction, these are also distinct cognitive processes performing specific functions, and individual differences have been observed on their performance (e.g., Reineberg et al., 2015). Nevertheless, our results should be interpreted with caution, as we did not control for practice effects as rigorously as we did for working memory.

For our fifth question, we evaluated other possible sources of bias in addition to practice effects, which were already analyzed in questions 1 and 2. In this case, we examined sources of bias considered in the RoB 2. In general, most studies presented the same concerns regarding the risk of bias with a few presenting a high risk or higher likelihood of biased results. We reanalyzed the data excluding these studies to look for any possible difference in the effect size, however we found it mostly remained unchanged. This could be due to the fact that most of the strengths and weakness found were shared across studies. For example, the inclusion of active control groups and the fact that these studies are not aiming to treat a health problem almost eliminates the risk of participants switching between groups as an attempt to receive a treatment, one of the factors assessed by the RoB 2. One other strength is the use of objective measures for the assessment in WM across all included studies, as this virtually eliminates biases in the scores produced by the examiners. However, we also found that most of the studies did not report how the random sequence used for group assignment was generated and did not pre-register the instruments and analysis plan. This is a problem since many authors use the term ‘random’ to describe procedures that are not truly random (Sterne et al., 2019) and the lack of pre-registration of instruments and analyses gives researchers the opportunity to choose to report the instruments and analyses that are most convenient to their interests. It is important to bear in mind that this is a controversial field with strong points of view and commercial interests involved. These factors could lead researchers to practices that produce bias, such as allocating participants to groups in a nonrandom fashion or performing different analyses that “facilitate” the occurrence of the most favourable results for the research team. We therefore recommend that authors investigating CCT should reduce the risk of bias in their studies by using and reporting proper random allocation procedures for their samples and by pre-registering their studies.

For our final question, we examined the influence of various methodological choices on the outcomes of cognitive training programmes. After the matter of practice effects, the most controversial methodological issue in this field arguably concerns the type of control group used for comparison. Active control groups have been recommended over passive controls on the basis that the former exert placebo effects, something that cannot be controlled with passive groups (Melby-Lervåg & Hulme, 2016). However, it is not clear to what extent this is true, and it is not uncommon to find studies including both type of control groups (Maraver et al., 2016; Redick et al., 2013; Ward et al., 2017) which allows comparison of effects. For example, the study by Maraver et al. (2016) found that the effects of training on some cognitive measures were only significant when comparing the treatment group with the passive control group. To investigate this, we performed separate analyses comparing CCT groups with active and passive controls. We found that the effect sizes were very similar for both type of groups. Au et al. (2020) addressed this issue where results from two studies are reported. The first study involved a meta-meta-analysis—that is, a meta-analysis of meta-analyses—and the second study was a meta-analysis including articles that used both types of control group. In both cases, effect sizes produced by either type of control were compared and their results indicated that, although passive controls tend to produce larger effect sizes, these differences might not be meaningful enough to claim that passive control groups should not be used. Although some authors (Gobet & Sala, 2022) have observed several limitations in the study by Au et al. (2020), such as violations of the assumption of independence, the exclusion of studies published after 2016, or the way in which active controls were defined, our review did not find sufficient evidence to support for the use of active control over passive control groups.

We also performed a set of meta-regressions to evaluate the role of specific characteristics of the studies in the effect size, namely, the number of participants, the time dedicated to training, where the training programme was delivered (home or laboratory), and the type of training (i.e., single-task, multitask, and multiprocess). None of these variables moderated the effect of training. Our results are consistent with those found in other meta-analyses (Melby-Lervåg et al., 2016; Soveri et al., 2017) where no effects were found in any of the moderator variables they analyzed. These results seem counterintuitive, particularly the lack of moderator effects from dosage and type of training. It would be expected, and it is commonly believed, that dosage plays an important role in the effects of training (e.g., Klingberg, 2010; Schmiedek et al., 2010), and that programmes targeting multiple processes with different tasks have a better chance of generalizing their effects to other areas (e.g., Klingberg et al., 2002; Ward et al., 2017). The present analysis does not provide support for this assertion. However, it is important to note that the minimum number of sessions found in the studies included in this review was six, and the most common training programme used in the category of single-task studies was the *n*-back training. It is possible that a relatively small number of sessions using a complex cognitive task, such as the *n*-back task that requires EF for its performance (Gajewski et al., 2018; Mencarelli et al., 2019), may be sufficient to produce small improvements in WM. It is important to note that despite the relatively small number of participants in most of the studies, we found no evidence of a negative impact on the results in the meta-regressions. Similarly, the funnel plot, which is instrumental in identifying small-study effects, also did not indicate any adverse impacts.

In summary, CCT seems to have a positive impact on cognition with healthy adults, though the effect sizes may be too small to result in meaningful real-world change. However, we also identified several potential sources of bias in the field that may bias the reported results, such as the methods used to allocate participants to experimental and control groups and the lack of study pre-registration. Furthermore, the choice of assessment instruments seems to have the strongest impact. Our rigorous approach to isolating practice effects resulted in smaller effect sizes than those reported in prior meta-analyses (Kelly et al., 2014; Leung et al., 2015; Motter et al., 2016; Sitzer et al., 2006). In contrast, our analyses of practice effects produced very large effect sizes. This underscores the discussion about the impact of practice effects, a problem for which no single solution has yet been provided. Our method offers valuable insights into the transferability of training effects, since we required the trained and assessment task to employ both different stimuli and different types of task. If improvements are mainly due to practice effects, future studies should closely examine which elements are common to tasks from different paradigms, such as *n*-back tasks and complex span tasks. Thus, while our findings point toward modest improvements in cognitive functioning through CCT, they also raise critical questions about the reliability and interpretation of such effects.

## Supplementary Information

Below is the link to the electronic supplementary material.

Supplementary file1 (DOCX 850 KB)
