# Supplementary material for: Can we enhance working memory? Bias and effectiveness in cognitive training studies
Source: Psychon Bull Rev. 2024 Feb 16;31(5):1891–914. doi: 10.3758/s13423-024-02466-8 (PMC11543728; doi:10.3758/s13423-024-02466-8)

**Supplemental Materials**

**S1. Questions in the risk of bias assessment tool**

***Domain 1: Bias arising from the randomization process***

1.1 Was the allocation sequence random?

1.2 Was the allocation sequence concealed until participants were enrolled and assigned to interventions?

1.3 Did baseline differences between intervention groups suggest a problem with the randomization process?

***Domain 2: Bias due to deviations from intended interventions***

2.1 Were participants aware of their assigned intervention during the trial?

2.2 Were carers and people delivering the interventions aware of participants' assigned intervention during the trial?

If Yes, Probably Yes, or No Information to 2.1 or 2.2:

2.3 Were there deviations from the intended intervention that arose because of the experimental context?

If Yes or Probably Yes to 2.3:

2.4 Were these deviations likely to have affected the outcome?

If Yes, Probably Yes, or No Information to 2.4:

2.5. Were these deviations from intended intervention balanced between groups?

2.6 Was an appropriate analysis used to estimate the effect of assignment to intervention?

If No, Probably No, or No Information to 2.6:

2.7 Was there potential for a substantial impact (on the result) of the failure to analyse participants in the group to which they were randomized?

***Domain 3: Bias due to missing outcome data***

3.1 Were data for this outcome available for all, or nearly all, participants randomized?

If No, Probably No, or No Information to 3.1:

3.2 Is there evidence that result was not biased by missing outcome data?

If No or Probably No to 3.2:

3.3 Could missingness in the outcome depend on its true value?

If Yes, Probably Yes, or No Information to 3.3:

3.4 Is it likely that missingness in the outcome depended on its true value?

***Domain 4: Bias in measurement of the outcome***

4.1 Was the method of measuring the outcome inappropriate?

4.2 Could measurement or ascertainment of the outcome have differed between intervention groups?

4.3 Were outcome assessors aware of the intervention received by study participants?

If Yes, Probably Yes, or No Information to 4.3:

4.4 Could assessment of the outcome have been influenced by knowledge of intervention received?

If Yes, Probably Yes, or No Information to 4.4:

4.5 Is it likely that assessment of the outcome was influenced by knowledge of intervention received?

***Domain 5: Bias in selection of the reported result***

5.1 Were the data that produced this result analysed in accordance with a pre-specified analysis plan that was finalized before unblinded outcome data were available for analysis?

5.2 Is the numerical result being assessed likely to have been selected, on the basis of the results, from multiple eligible outcome measurements (e.g. scales, definitions, time points) within the outcome domain?

5.3 Is the numerical result being assessed likely to have been selected, on the basis of the results, from multiple eligible analyses of the data?

**S2. Risk of Bias assessment for each study**

**
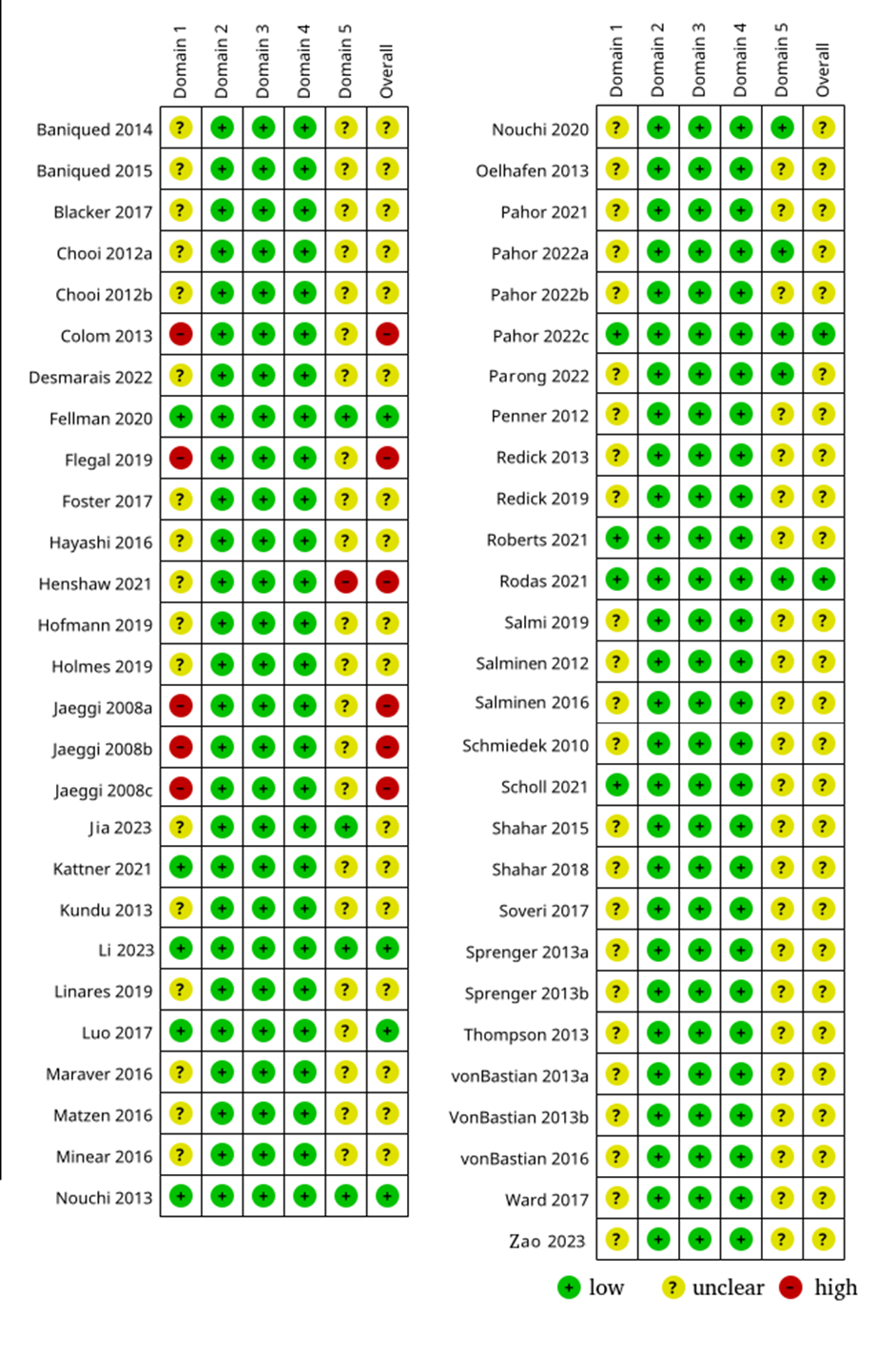
**

**Figure S3**

**Forestplot from the effects of training on Processing Speed**


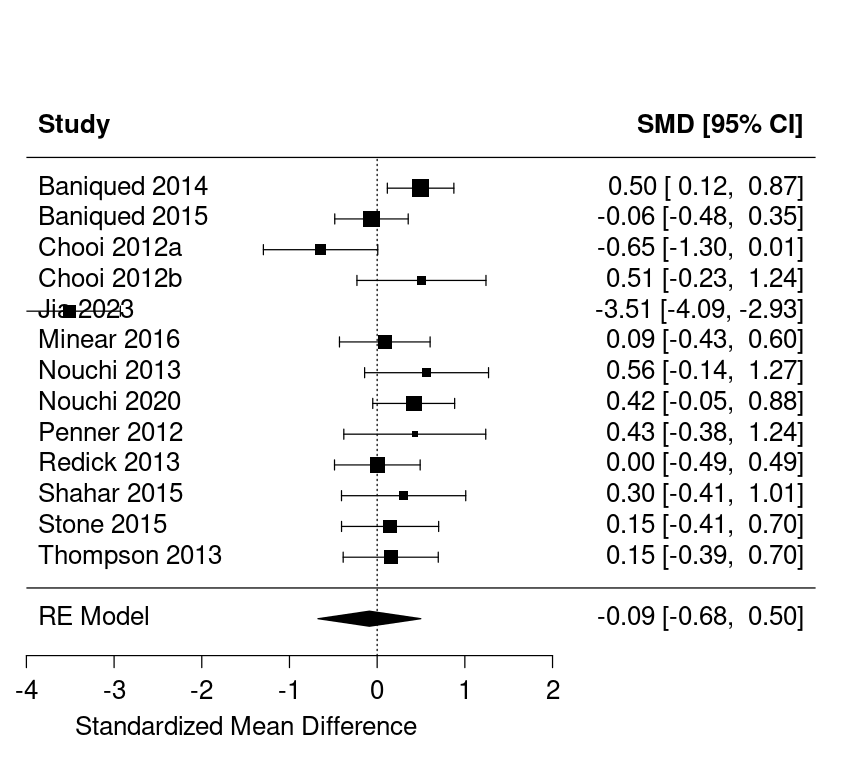


**Figure S4**

**Forestplot from the effects of training on Short term memory**


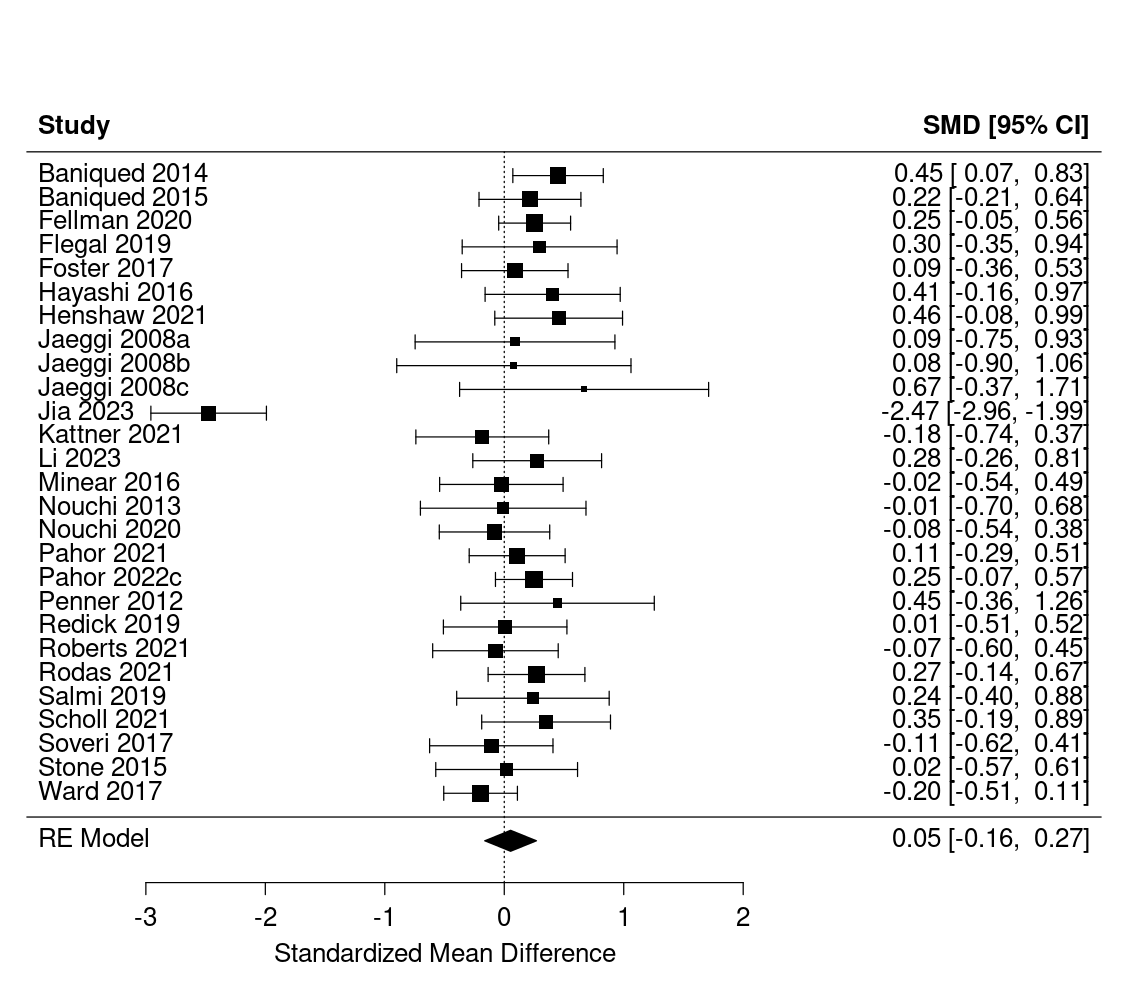

Supplement: Supplementary file 1 — Supplementary file1 (DOCX 850 KB) [file 13423_2024_2466_MOESM1_ESM.docx]
